# Spherization indices measured by resting SPECT improve risk stratification in patients with ischemia with non-obstructive coronary artery disease (INOCA)

**DOI:** 10.1186/s13550-024-01075-9

**Published:** 2024-02-07

**Authors:** Yuting Zhao, Yingqi Hu, Yuanyuan Li, Yanhui Wang, Yuxin Xiao, Li Xu, Tailin Ren, Qiuyan Wu, Ruonan Wang, Zhifang Wu, Sijin Li, Ping Wu

**Affiliations:** 1 Department of Nuclear Medicine, First Hospital of Shanxi Medical University, Taiyuan, China; 2 Collaborative Innovation Center for Molecular Imaging of Precision Medicine, Shanxi Medical University, Taiyuan, China; 3 Shanxi Key Laboratory of Molecular Imaging, Shanxi Medical University, Taiyuan, Shanxi China

**Keywords:** Left ventricular spherical indices, INOCA, Prognosis, SPECT

## Abstract

**Background:**

The prevalence of ischemia with non-obstructive coronary artery disease (INOCA) is substantial, but its risk stratification has been suboptimal. Resting SPECT myocardial perfusion imaging (MPI) could provide useful heart information including spherical indices. We aimed to evaluate the prognostic value of spherical indices in individuals with INOCA.

**Results:**

During a median follow-up of 47.2 ± 20.8 months, 49 (17.2%) patients experienced major adverse cardiac events (MACE). Compared to those without MACE, those with MACE had a higher shape index (SI) (0.60 ± 0.07 vs. 0.58 ± 0.06; *P* = 0.028) and a lower E2 (eccentricity index calculated by the QPS) (0.81 ± 0.05 vs. 0.83 ± 0.04; *P* = 0.019). MACE event-free survival analysis revealed significant differences in the SI and E2 among all patients (all log-rank *P* < 0.01). Multivariate Cox analysis showed abnormal SI (HR: 2.73, 95% CI 1.44–5.18, *P* = 0.002) and E2 (HR: 1.94, 95% CI 1.08–3.48, *P* = 0.026) were both independent predictors for MACE when they were put into the same model, respectively. The incorporation of the SI into the baseline model demonstrated a significant improvement in the predictive accuracy for MACEs (*P* = 0.026), whereas E2 did not exhibit a similar improvement (*P* > 0.05).

**Conclusion:**

For patients with INOCA, spherical indices (especially the SI) were associated with long-term MACE, which could be a preferable indicator for risk stratification and prognostic prediction.

## Background

Ischemia with non-obstructive coronary artery disease (INOCA) is a perplexing condition that affects approximately 50–70% of patients who experience chest pain and detectable myocardial ischemia [1]. Recent studies have shed light on the complexity and variability of INOCA, emphasizing its heterogeneous nature and its link to a higher risk of major adverse cardiac events (MACE) and all-cause mortality [2, 3]. Different subtypes of INOCA have been identified, including coronary microvascular dysfunction (CMD) (including endothelial dysfunction) and epicardial coronary artery spasm [4]. This increased risk underscores the importance of awareness and early management of INOCA to improve patient outcomes.

Myocardial perfusion imaging (MPI) with single photon emission computed tomography (SPECT) has emerged as an invaluable tool in the field of clinical cardiology. One of its most significant advantages is its ability to provide a comprehensive and multidimensional evaluation of the heart [5–7]. A holistic understanding of the patient's cardiovascular status can be obtained by assessing not only myocardial perfusion but also wall motion, cardiac function, and remodeling. Although left ventricular remodeling (LVR) is widely known to be associated with MACEs, there is still a lack of information on the prognostic value of LVR variables in MPI [8–10]. The majority of the research being conducted on SPECT spherical indices has focused on stress MPI, and additional research is needed to determine which of these parameters is best for clinical practice. In addition, although stress MPI continues to be the preferred approach in most cases, resting SPECT MPI serves as a valuable adjunct in situations where stress testing is contraindicated or not feasible [5].

Consequently, investigating the independent and incremental prognostic value of resting SPECT MPI spherical indices for MACE classification in patients with INOCA is the primary objective of the present study.

## Methods

### Study population

A total of 6386 consecutive patients with resting SPECT MPI were analyzed retrospectively between January 2016 and March 2021 at the First Hospital of Shanxi Medical University, in which 1909 patients were followed. The inclusion criteria for patients were as follows: (1) diagnosed or suspected of having coronary artery disease (CAD); (2) had echocardiographic left ventricular ejection fraction (LVEF) within three months; (3) had no structural heart disease, cancer, respiratory failure, severe liver or kidney disease, severe infection, or other disease that may significantly affect the LVEF; and (4) had analyzable imaging data. The remaining patients were excluded if they had (1) a previous history of cardio-cerebrovascular disease, percutaneous coronary intervention (PCI), or coronary artery bypass graft (CABG) (*n* = 856); (2) coronary artery stenosis ≥ 50% or missing (*n* = 545); (3) no follow-up results (*n* = 183); or (4) obstructive CAD and acute myocardial infarction (*n* = 40). Each patient was informed of the purpose of the study and provided signed informed consent. This study conformed to the Declaration of Helsinki and was approved by the institutional ethics committee of the First Hospital of Shanxi Medical University (ID 2022-K-128). Here, INOCA was defined as the presence of signs and symptoms associated with myocardial ischemia, including typical angina, atypical angina, or nonanginal chest pain; these signs and symptoms were correlated with ischaemic alterations observed by electrocardiogram and/or MPI and without detectable coronary stenosis or luminal stenosis < 50% on invasive coronary angiography [1–3].

### Clinical data

A comprehensive set of demographic and clinical information, laboratory serological biomarker and echocardiography data was collected. The demographic and clinical information consisted of clinical history, age, sex, body mass index (BMI), as well as cardiovascular risk factors such as smoking, diabetes, hypertension, and dyslipidemia; family history of CAD; clinical symptoms; abnormal electrocardiogram changes; drug use; and coronary artery stenosis grade. The laboratory indices included complete blood count, inflammation, and metabolism (fasting blood glucose, hemoglobin, cholesterol, triglycerides, low-density lipoprotein, high-density lipoprotein, and homocysteine). The echocardiography data obtained within three months intervals from the MPI, included the LVEF, end diastolic volume (EDV), and end systolic volume (ESV).

### Acquisition of resting MPI

The imaging protocol for all patients comprised gated resting SPECT MPI using a single IQ-SPECT double probe scanner (Symbia T16, Siemens Medical System, Germany). ^99m^Tc-methoxyisobutylisonitrile (MIBI) (740–925 MBq) was injected intravenously after the patients had fasted for at least 4 h. Fatty meals were applied for 15–20 min before imaging. Patients remained in a supine position with their arms raised and fixed in the support groove. The acquisition was initiated 60 min after the injection. The acquisition speed was 25 s/frame, and the process lasted 8 min with the 208° rotary step method, 34 frames, and 6°/frame. Images were reconstructed by the method of ordered subset expectation maximization (OSEM). All acquisitions were conducted in a fully automated mode.

### Quantification analyses of the MPI

Quantification parameters, including perfusion-related total perfusion deficit (TPD) and summed rest score (SRS), remodeling-related shape index (SI), end diastolic shape index (EDSI), end systolic shape index (ESSI), eccentricity index (EI), EDV, and ESV, function-related LVEF, peak ejection rate (PER), peak filling rate (PFR), and time to PFR (TTPF), as well as motion/systolic synchrony-related summed motion score (SMS), summed thickening score (STS), bandwidth (BW), standard deviation (SD), and entropy were obtained automatically by quantitative gated SPECT (QGS) and quantitative perfusion SPECT (QPS) software (Cedars-Sinai Medical Center, Los Angeles, CA). The EDSI and ESSI were defined as the ratio of the maximum transverse diameter of the short axis to the diameter of the long axis at the end diastolic and end systolic, respectively. The values ranged from 0 to 1, where the higher the value was, the more spherical the heart became. The EI was an index reflecting LV elongation, with a value ranging from 0 to 1, the lower the EI was, the more spherical the heart became. E1 and E2 were defined as the EI calculated by QGS and QPS, respectively.

### Follow-up

The primary endpoints were composite MACE for cardiovascular death, nonfatal MI, late (> 90 days after SPECT MPI) revascularization (including PCI or CABG), and readmission for heart failure or unstable angina. For each person, the most severe condition was recorded to determine the occurrence of MACEs. The follow-up period for all patients took place from April to August 2022. Multiple methods were employed to ensure comprehensive and accurate information, including an electronic medical records system, phone calls to patients or their relatives, or consultation from their referring physician. The duration of follow-up was measured as the interval between the date of SPECT imaging and the occurrence of the first MACE. Early revascularization was not regarded as a MACE.

### Statistical analysis

Patients were categorized into groups based on the occurrence of MACE, and the continuous variables are expressed as the mean ± standard deviation (SD); categorical data are presented as the frequency and percentage. Student’s t test or the Mann–Whitney U test was used to compare continuous variables according to the test results of a normal distribution and homogeneity of variance, while the χ2 test was used to compare categorical variables. To explore the relationships among all spherical indices, Pearson correlation coefficients were calculated.

All variables were initially evaluated using univariate Cox regression analysis. The spherical indices were considered both continuous and binary variables (using the optimal cut-off value obtained through restricted cubic splines). Variables with statistical significance, including clinical characteristics and MPI findings, were entered into a multivariate Cox regression model to identify predictors of MACEs. To address the issue of multicollinearity, a separate model was created for each spherical variable after adjusting for other factors as the baseline model. The event-free survival curve was generated by the Kaplan–Meier method and compared with the log-rank test.

We established the continuous net reclassification improvement (NRI), categorized the NRI and integrated discrimination improvement (IDI) and used the 5-year follow-up period as the reference point for these calculations to assess the incremental prognostic value of the spherical indices and their impact on risk prediction. Researchers could evaluate the added value of the variables in improving risk prediction accuracy by performing these reclassification analyses.

All statistical analyses were performed in R language version 4.3.0 (http://www.R-project.org), and a *P* value < 0.05 (two-sided) was considered to indicate statistical significance.

## Results

### Patient characteristics

A total of 285 INOCA patients (mean age 56.6 ± 12.3 years, 129 women) were enrolled and followed for 47.2 ± 20.8 months (range 5.8–80.3 months). Among them, 49 (17.2%) patients experienced MACE, of which 46 (16.1%) had readmissions, 1 (0.4%) had late revascularization, 1 (0.4%) had nonfatal MI, and 1 (0.4%) had cardiovascular death.

After the follow-up, patients were categorized into two groups based on their outcomes: MACE and non-MACE. Table 1 provides a comprehensive summary of the baseline characteristics of the patients. All patients presented with symptoms of myocardial ischemia. In addition, 8 (2.8%) individuals also experienced gastrointestinal distress, including indigestion, nausea and vomiting.

**Table 1:** Characteristics of enrolled patients according to MACE

	Total (*N* = 285)	MACE (*N* = 49)	Non-MACE (*N* = 236)	*P* value
Age (years)	56.6 ± 12.3	61.2 ± 11.5	55.7 ± 12.2	0.004
Women, *n(%)*	129 (45.3)	24 (49.0)	105 (44.5)	0.566
BMI (kg/m^2^)	24.7 ± 3.6	23.9 ± 3.4	24.8 ± 3.6	0.096
Clinical status				
Typical angina, *n(%)*	144 (50.5)	26 (53.1)	118 (50.0)	0.821
Atypical angina, *n(%)*	98 (34.4)	17 (34.7)	81 (34.3)	
Non-anginal chest pain, *n(%)*	43 (15.1)	6 (12.2)	37 (15.7)	
Abnormal electrocardiogram, *n(%)*	170 (59.6)	27 (55.1)	143 (60.6)	0.476
Coronary artery stenosis				
Without detectable stenosis, *n(%)*	166 (58.2)	21 (42.9)	145 (61.4)	0.055
1% ~ 24%, *n(%)*	74 (26.0)	17 (34.7)	57 (24.2)	
25% ~ 49%, *n(%)*	45 (15.8)	11 (22.4)	34 (14.4)	
Medical history				
Hypertension, *n(%)*	128 (44.9)	26 (53.1)	102 (43.2)	0.208
Diabetes, *n(%)*	38 (13.3)	10 (20.4)	28 (11.9)	0.109
Smoking, *n(%)*	103 (36.1)	17 (34.7)	86 (36.4)	0.817
Dyslipidemia, *n(%)*	17 (6.0)	4 (8.2)	13 (5.5)	0.475
Cardiovascular family history, *n(%)*	92 (32.3)	20 (40.8)	72 (30.5)	0.160
Medications				
Anticoagulant/Antiplatelet, *n(%)*	219 (76.8)	41 (83.7)	178 (75.4)	0.213
ACEI/ARB, *n(%)*	113 (39.6)	20 (40.8)	93 (39.4)	0.854
Beta-blocker, *n(%)*	123 (43.2)	19 (38.8)	104 (44.1)	0.496
CCB, *n(%)*	83 (29.1)	20 (40.8)	63 (26.7)	0.048
Statin, *n(%)*	212 (74.4)	41 (83.7)	171 (72.5)	0.102
Laboratory variables				
Hemoglobin *(g/L)*	142.4 ± 15.0	137.0 ± 16.0	143.5 ± 14.6	0.006
Red blood cell *(*× *10*^*12*^*/L)*	4.6 ± 0.9	4.4 ± 0.5	4.7 ± 0.9	0.019

ACEI, angiotensin converting enzyme inhibitors; ARB, angiotensin receptor blockers

Patients who experienced MACEs were older (61.2 ± 11.5 vs. 55.7 ± 12.2, *P* = 0.004) and had lower hemoglobin (137.0 ± 16.0 g/L vs. 143.5 ± 14.6 g/L, *P* = 0.006), red blood cells counts (4.4 × 10^12^/L vs. 4.7 × 10^12^/L, *P* = 0.019), and higher calcium channel blocker (CCB) use (40.8% vs. 26.7%, *P* = 0.048). All other baseline characteristics did not differ between the groups.

### Patient imaging findings according to MACEs

**Fig. 1:**
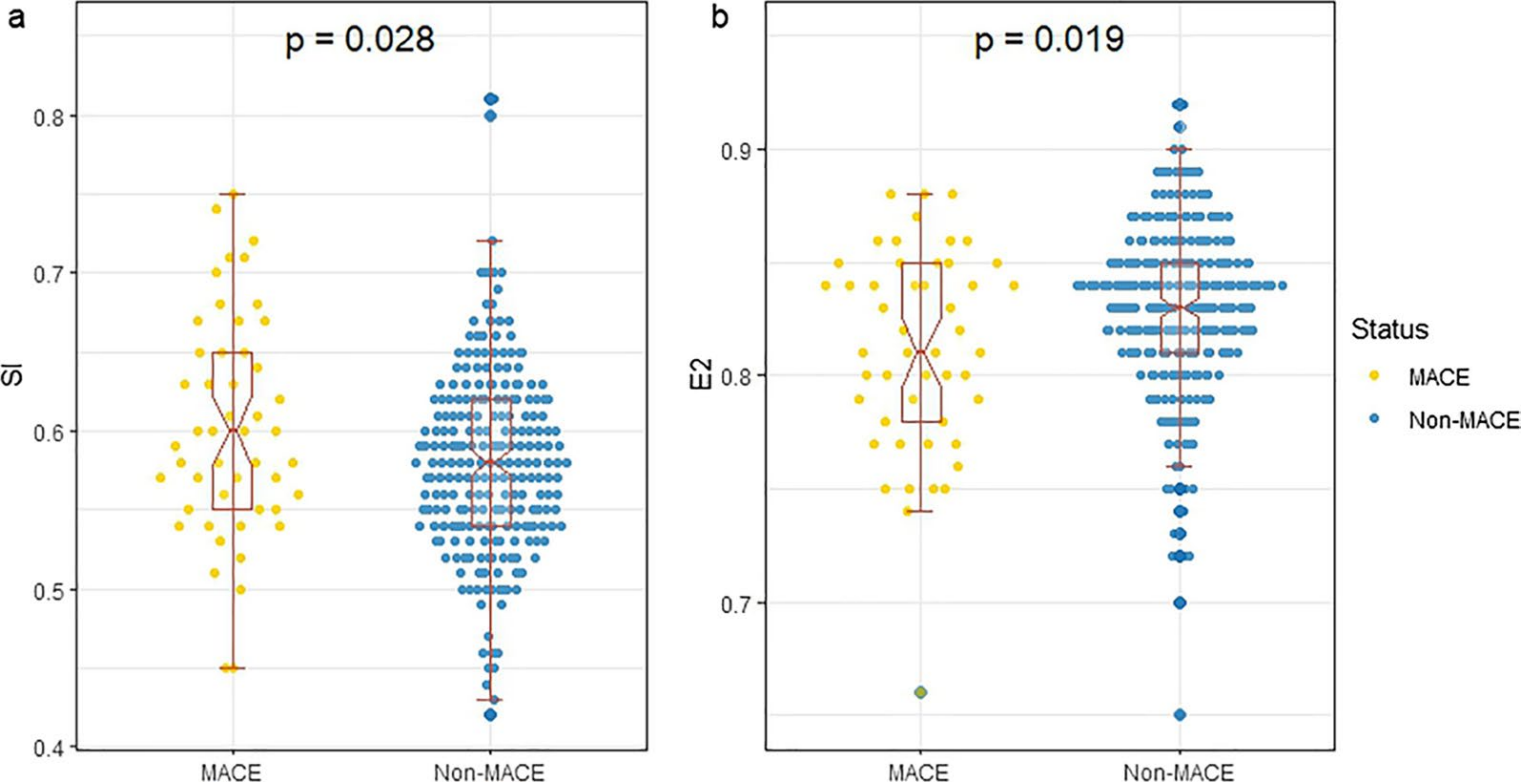
Patients with MACEs had abnormal spherical indices. Patients with MACEs had abnormal spherical indices, including higher SI (**a**) and lower E2 (**b**) (all *P* < 0.05)

**Fig. 2:**
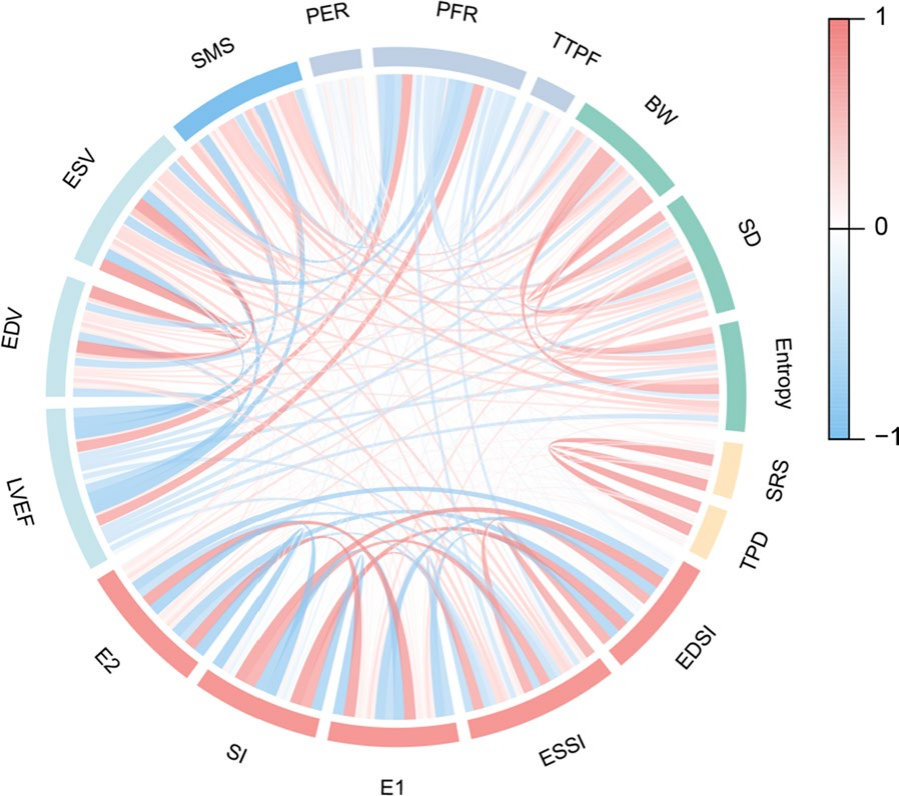
The correlation between spherical indices and conventional parameters. Strong correlations were observed among each pair of spherical indices (all *P* < 0.001).

Table 2 provides the echocardiography and MPI results for the patients. Patients with MACE had lower -PER (1.60 ± 0.62 vs. 1.82 ± 0.67, *P* = 0.034), greater TTPF (271.78 ± 45.15 vs. 255.29 ± 44.77, *P* = 0.020), and abnormal spherical indices, including higher SI (0.602 ± 0.070 vs. 0.580 ± 0.061, *P* = 0.028) and lower E2 (0.812 ± 0.045 vs. 0.828 ± 0.042, *P* = 0.019) (Fig. 1). No differences in the other indicators were observed. Strong correlations were observed among each pair of spherical indices (all *P* < 0.001, Fig. 2). The thresholds of SI and E2 were 0.65 and 0.81, respectively.

**Table 2:** Patients’ imaging findings according to MACE

	Total (*N* = 285)	MACE (*N* = 49)	Non-MACE (*N* = 236)	*P* value
Echocardiography				
LVEF *(%)*	63.77 ± 9.33	64.06 ± 10.42	63.71 ± 9.11	0.812
LVEDV *(mL)*	114.66 ± 35.98	110.40 ± 28.71	115.52 ± 37.26	0.402
LVESV *(mL)*	42.12 ± 24.81	38.17 ± 15.89	42.91 ± 26.19	0.259
Parameters of QGS				
LVEF *(%)*	50.80 ± 10.36	49.84 ± 10.24	51.00 ± 10.39	0.474
LVEDV *(mL)*	83.21 ± 38.83	79.98 ± 31.57	83.88 ± 40.20	0.524
LVESV *(mL)*	43.85 ± 32.91	42.57 ± 27.66	44.11 ± 33.94	0.766
SMS	10.88 ± 9.80	11.71 ± 9.92	10.70 ± 9.79	0.512
STS	6.15 ± 6.61	5.84 ± 6.63	6.21 ± 6.62	0.719
EDSI	0.624 ± 0.061	0.637 ± 0.062	0.621 ± 0.060	0.111
ESSI	0.500 ± 0.070	0.516 ± 0.074	0.497 ± 0.070	0.087
EI	0.842 ± 0.046	0.834 ± 0.044	0.844 ± 0.046	0.153
-PER *(EDV/s)*	1.78 ± 0.67	1.60 ± 0.62	1.82 ± 0.67	0.034
PFR *(EDV/s)*	1.89 ± 0.54	1.77 ± 0.50	1.91 ± 0.55	0.090
TTPF *(ms)*	258.12 ± 45.19	271.78 ± 45.15	255.29 ± 44.77	0.020
BW *(°)*	61.43 ± 39.83	59.27 ± 35.45	61.88 ± 40.74	0.676
SD *(°)*	20.78 ± 10.78	20.56 ± 10.05	20.82 ± 10.94	0.874
Entropy *(%)*	43.32 ± 9.10	43.73 ± 9.25	43.23 ± 9.08	0.726
Parameters of QPS				
SRS	5.39 ± 4.48	5.29 ± 4.07	5.42 ± 4.57	0.854
TPD *(%)*	6.62 ± 6.01	6.35 ± 5.35	6.68 ± 6.15	0.726
SI	0.584 ± 0.063	0.602 ± 0.070	0.580 ± 0.061	0.028
EI	0.825 ± 0.042	0.812 ± 0.045	0.828 ± 0.042	0.019

**Fig. 3:**
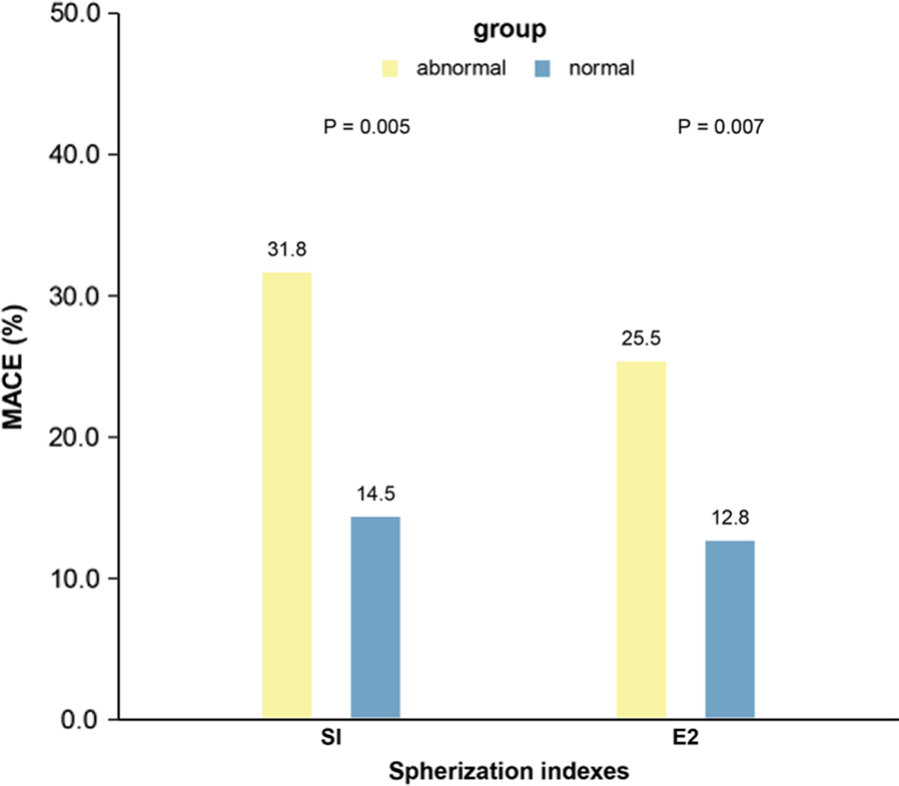
MACEs occurrence between different spherical indices for INOCA patients. INOCA patients with abnormal SI and E2 occurred more MACEs compared to those with normal indices (all *P* < 0.05)

Among the enrolled patients, 44 (15.4%) and 98 (34.4%) had abnormal SI and E2, respectively. Among these patients, 14 (31.8%) and 25 (25.5%) had MACE (Fig. 3). The results depicted in Fig. 3 reveal significant differences in the occurrence of MACEs between patients with abnormal SI and E2 and those with normal indices.

### Multivariate Cox analyses of the sphericity indices for MACE

According to the univariate Cox regression analysis, when the spherization indices were included as continuous variables, the ESSI, SI and E2 were found to be significant. When transformed into binary variables based on the optimal cut-off values, the SI and E2 remained meaningful (all *P* < 0.05). To account for potential confounding factors, further adjustments were made for age, coronary artery stenosis, CCB, hemoglobin, -PER, TPD and TTPF in the multivariate Cox regression analysis. The results demonstrated that these two spherization indices remained significant independent predictors, indicating their robust association with MACEs (Table 3).

**Table 3:** Unadjusted and adjusted HRs for MACE by categorized variables

	Unadjusted HR (95%CI)	*P* value	Adjusted HR (95%CI)	*P* value
SI	2.42 (1.30–4.49)	0.005	2.73 (1.44–5.18)	0.002
E2	2.21 (1.26–3.86)	0.006	1.94 (1.08–3.48)	0.026

### Incremental prognostic value of spherization indices

The prediction accuracy for MACEs was significantly improved when SI (continuous NRI [95% CI], 0.483 [− 0.017–0.320], *P* = 0.066; categorical NRI [95% CI], 0.029 (− 0.065–0.122), *P* = 0.546; IDI [95% CI], 0.039 (0.005–0.103), *P* = 0.026) was included in the model in comparison with E2 (Table 4).

**Table 4:** NRI and IDI analysis for spherization indices in MACE prediction

	Continuous NRI (95% CI)	*P* value	Categorized NRI (95% CI)	*P* value	IDI (95% CI)	*P* value
Baseline	Baseline		Baseline		Baseline	
Baseline + SI	0.148 (− 0.017–0.320)	0.066	0.029 (− 0.065–0.122)	0.546	***0.039 (0.005–0.103)***	***0.026***
Baseline + E2	0.170 (− 0.086–0.303)	0.212	0.031 (− 0.043–0.109)	0.394	0.023 (− 0.001–0.067)	0.093

Bolditalic is to emphasis that the prediction accuracy for MACEs was significantly improved when SI was included in the model in comparison with E2

Baseline include age, coronary artery stenosis, CCB, hemoglobin, PER, TPD and TTPF

### K-M analysis

**Fig. 4:**
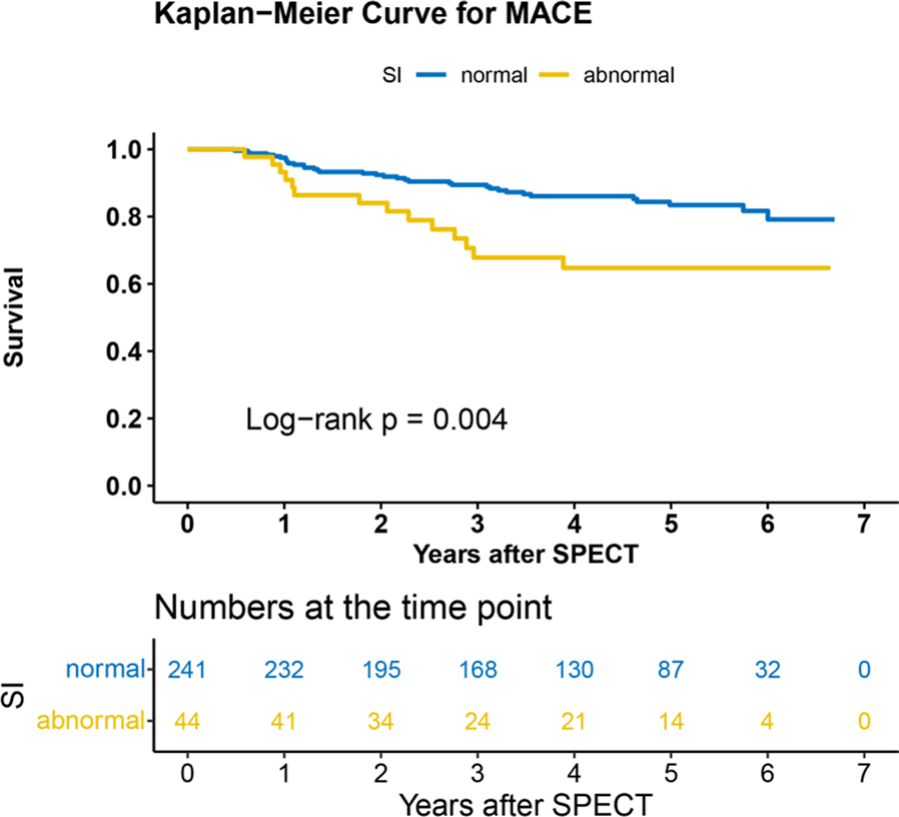
Survival analysis of the SI in INOCA patients. The event-free survival rate for MACEs was significantly lower in patients with abnormal SI compared to those with normal SI

In the entire cohort, the event-free survival rate for patients with abnormal SI was significantly lower than that for patients with a normal index (log-rank = 9.162, *P* = 0.003, Fig. 4). A similar result was observed for E2 (log-rank = 8.068, *P* = 0.005).

### Visualization and evaluation of multivariate Cox regression analysis

**Fig. 5:**
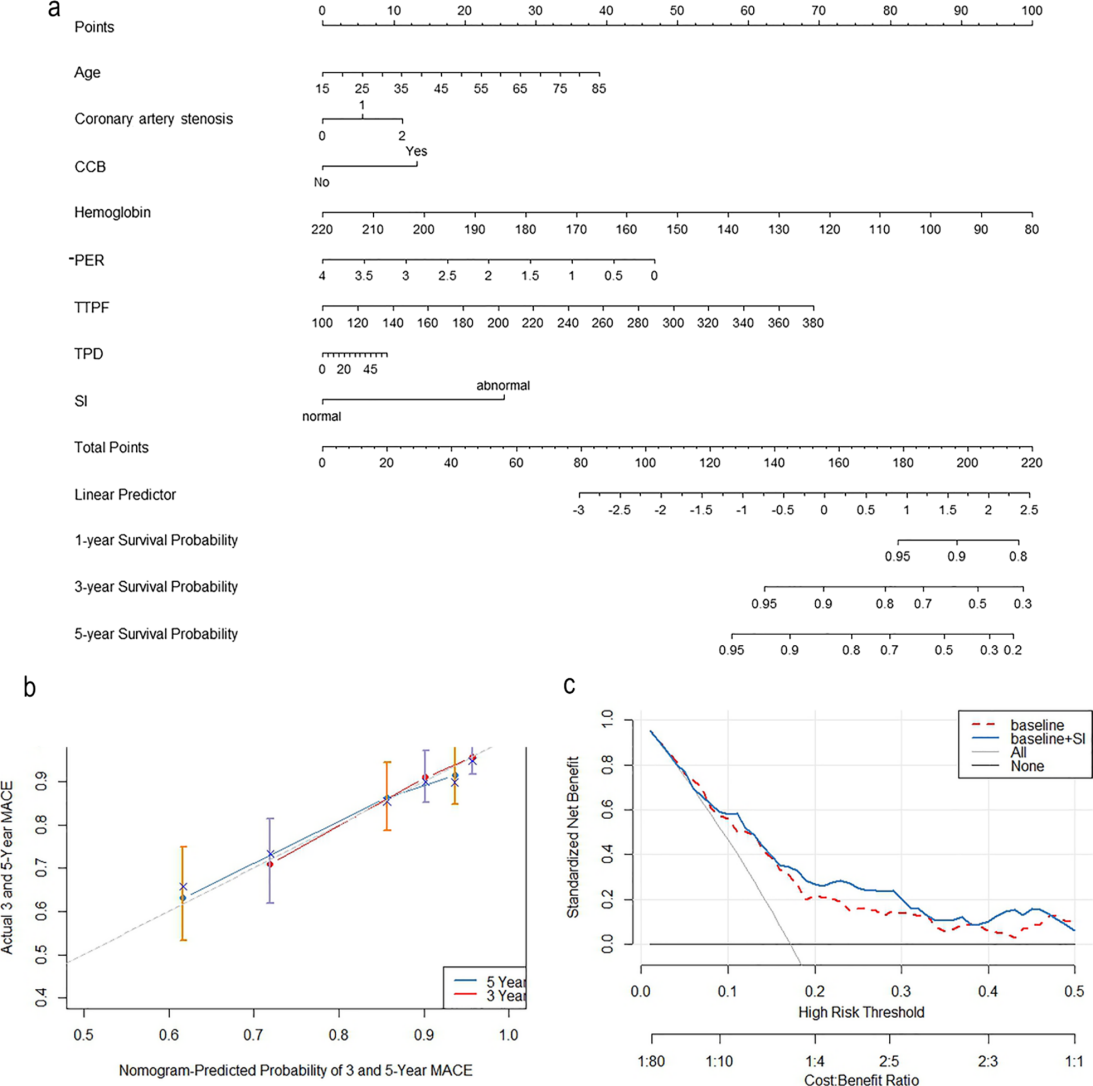
Visualization and evaluation of multivariate Cox regression analysis. Nomogram for predicting 1-, 3- and 5-year of survival probability (**a**), calibration curve for 3- and 5-year predict (**b**) and decision curve analysis for 5-year predict (**c**). Coronary artery stenosis, 0: without detectable stenosis; 1: 1% ~ 24%; 2: 25% ~ 49%

The nomogram utilized age, coronary artery stenosis, CCB, hemoglobin, -PER, TTPF, TPD and SI to estimate the risk of MACEs (Fig. 5a). The C-index was 0.724 (95% CI 0.684–0.764) and AUC values for 3- and 5-year MACE risk were 0.732 (95% CI 0.647–0.817) and 0.688 (95% CI 0.601–0.776), respectively. The calibration curve exhibited good agreement between the predicted and observed outcomes (Fig. 5b). The decision curve analysis indicated that the nomogram yielded a greater net benefit in predicting the risk of MACEs across a broader range of threshold probabilities (Fig. 5c).

## Discussion

Our study investigated for the first time whether the use of resting SPECT MPI LV spherical indices as a measurement method for LVR could be used to improve MACE prediction and provide incremental prognostic value in patients with INOCA. The main findings of this retrospective analysis are summarized as follows: (1) multiple spherical indices (especially the SI and EI) were independently associated with MACEs, regardless of whether they were unadjusted or adjusted for patients with INOCA; (2) among these, the SI might be the most promising prognostic indicator.

The EDSI, ESSI, SI, and EI were the primary variables used in the SPECT LV spherical analysis. Previous studies have demonstrated that spherical indices are beneficial for risk stratification and can accurately predict patient prognosis [8, 10–12]. By assessing the degree of LV sphericity, crucial clinical information was provided to assist health care professionals in predicting outcomes and devising appropriate treatment strategies. In the present study, we showed that resting spherical indices were independently related to MACEs and that the SI outperformed other indices in predicting these adverse cardiac events.

Among the clinical data, laboratory marker, medication, and SPECT MPI findings included in the multivariable Cox analysis, spherical indices (SI and E2) exhibited a significant association with MACEs. However, only the addition of the SI to those variables enhanced the discriminatory value and reclassification for predicting MACEs. Furthermore, our previous study showed that for CAD patients with preserved LVEF, an abnormal SI (≥ 0.65) calculated by the QPS might be a more promising indicator of stable capacity for prognostic stratification under different perfusion conditions. It is worth noting that the consistency with our prior research results allows us to speculate on the generalizability and reliability of these thresholds preliminarily. This approach would enhance the external validity of the study findings and provide clinicians with more reliable evidence when making diagnostic and treatment decisions.

These results are in agreement with those of previous studies. The results of a retrospective study involving 674 suspected CAD patients with normal myocardial perfusion and normal LVEF revealed that even in this situation, the assessment of the SI could still identify patients at high risk of early LVR and adverse cardiac events [9]. Similarly, in a gated cadmium-zinc-telluride (CZT) SPECT study, it was reported that abnormal EI was also associated with significant LVR and functional abnormalities [10]. Moreover, in a clinical study involving 14,016 patients, both the SI and EI were strongly associated with MACEs [11]. However, the optimal thresholds obtained in our study differ from those used in previous studies, which might be attributed to the heterogeneity of the population and variations in the acquisition protocol or processing software used. Notably, the EDSI and ESSI showed poor prognostic ability in this study, which was inconsistent with previous results. Nitta K et al. [13] demonstrated the effective performance of ESSI in the early identification of LVR in patients with normal perfusion. Additionally, Abidov et al. [8] reported that ESSI could serve as a clinical indicator for risk stratification in patients with congestive heart failure among 186 patients with a low likelihood of CAD. This discrepancy might be attributed to the relatively mild condition of the patients included in our study, as there were no significant abnormalities in LV systolic or diastolic function. Moreover, there was no evident significant difference in systolic or diastolic function between the MACE and non-MACE groups, which could be one of the reasons why these two indices did not exhibit good predictive ability for prognosis.

In addition, our study revealed that only the SI had perfect discriminatory value, which could be connected with the calculation methods used for various spherical indices [11]. The SI was measured by defining the endocardial boundary of the three-dimensional (3D) cardiac model, whereas the EI involved the iterative construction of a 3D ellipsoid model that best fit the mid-segment of the LV myocardium. In various cardiac diseases, including INOCA, the endocardium is more susceptible to the effects of afterload or thrombosis, and its function is more susceptible to impairment [14].

In addition to SPECT, various imaging modalities can be used to assess LVR. The results of a study that enrolled 244 women with symptoms of ischemia without obstructive CAD, absence of CMD, and preserved LVEF showed that elevated LV end diastolic pressure was strongly associated with increased LV mass-to-volume and decreased LV end diastolic volume index measured by magnetic resonance imaging (MRI), which suggested that the study of LVR is essential in the future [15]. Furthermore, in another study focusing on suspected INOCA and intermediate coronary flow reserve, female patients with a lower myocardial perfusion reserve index (MPRI) measured by MRI were shown to have a greater tendency toward adverse LVR and impaired LV diastolic function, providing further evidence of CMD in INOCA patients [14]. Choi et al. [16] showed that patients with INOCA who exhibited abnormal LV geometry, as evaluated by echocardiography, were associated with a poorer prognosis. However, the spherization indices and their optimal cut-off values were not different among the different modalities or software packages.

In summary, a meta-analysis involving 35,039 INOCA patients had higher rates of all-cause mortality and nonfatal MI than the general population, indicating the critical importance of accurate risk stratification and early identification of risk factors for INOCA patients in clinical practice [17]. Currently, the diagnosis relies mainly on anatomical assessment (coronary angiography), while the application of functional tests is limited [18]. LV cardiac remodeling has an impact on all cellular components of the heart, including cardiomyocytes, fibroblasts, endothelial cells, and leukocytes [4, 19]. Cardiomyocytes can potentially compromised contractile function, hypertrophy, or even cellular death [19]. When cardiac fibroblasts are activated, they can induce an excessive buildup of collagen and promote the development of fibrosis [19]. Dysfunction of microvascular endothelial cells triggers an increase in reactive oxygen species (ROS) generation, expedites leukocyte infiltration, and finally diminishes capillary density and tissue hypoxia [19]. An important pathological feature of INOCA is microvascular dysfunction in the coronary arteries, which is partly caused by structural remodeling of the microvessels [4, 20]. The prevalence of this dysfunction typically fluctuates between 26 and 54% [20]. Recent studies have indicated that left ventricular systolic dyssynchrony and perfusion parameters measured by CZT-SPECT could be used for risk stratification and prognosis prediction of INOCA [21–23]. Our research findings might complement the clinical decision-making of INOCA patients.

Since the calculation of spherical indices could be achieved automatically using MPI without requiring additional imaging time or exposing patients to additional radiation, MPI is a feasible tool for clinical use, as it provides additional prognostic or risk information.

### Study limitations

There are several limitations in the current study. First, the reliability of the conclusions of this study has yet to be confirmed, as the nature of a single-center retrospective study introduces certain limitations that should be acknowledged. However, further research, such as multi-center trials or longitudinal studies, is needed to validate and strengthen the reliability of the conclusions. Second, the conclusions of this study are specific to resting SPECT MPI and do not encompass the evaluation of individuals under stress conditions. Further studies incorporating dynamic stress protocols (MPI) will be necessary to determine the generalizability of the results to individuals undergoing physiological stress. Third, due to the differences in software and imaging principles among the various modalities, the thresholds determined in the present study are currently applicable only to SPECT MPI.

## Conclusion

For patients with INOCA, spherical indices (especially the SI) were associated with long-term MACE, which could be a preferable indicator for risk stratification and prognostic prediction.

## Data Availability

The datasets generated and analyzed during this study is available from the corresponding author on reasonable request.

## Abbreviations

CAD: Coronary artery disease
CMD: Coronary microcirculation disorder
EDSI: End diastolic shape index
EI: Eccentricity index
ESSI: End systolic shape index
INOCA: Ischemia with non-obstructive coronary artery disease
LVEF: Left ventricular ejection fraction
LVR: Left ventricular remodeling
MACE: Major adverse cardiac events
MPI: Myocardial perfusion imaging
SI: Shape index
SPECT: Single photon emission computed tomography
